# ‘All the stars were aligned’? The origins of England’s National Institute for Health Research

**DOI:** 10.1186/s12961-019-0491-5

**Published:** 2019-12-04

**Authors:** Paul Atkinson, Sally Sheard, Tom Walley

**Affiliations:** Department of Public Health and Policy, University of Liverpool, Whelan Building, Quadrangle, Liverpool, L69 3GB United Kingdom

**Keywords:** United Kingdom, health research system, National Health Service, National Institute for Health Research

## Abstract

**Background:**

In 2006, the research and development (R&D) activity of England’s national healthcare system, the National Health Service, was reformed. A National Institute for Health Research (NIHR) was established within the Department of Health, the first body to manage this activity as an integrated system, unlocking significant increases in government funding. This article investigates how the NIHR came to be set up, and why it took the form it did. Our goal was a better understanding of ‘how we got here’.

**Methods:**

We conducted oral history interviews with 38 key witnesses, held a witness seminar, and examined published and unpublished documents.

**Results:**

We conclude that the most important forces shaping the origin of NIHR were the growing impact of evidence-based medicine on service policies, the growth of New Public Management ways of thinking, economic policies favouring investment in health R&D and buoyant public funding for healthcare. We note the strong two-way interaction between the health research system and the healthcare system — while beneficial for the use of research, challenges for healthcare (such as stop-go funding) could also produce challenges for health research.

**Conclusions:**

Understanding how and why England came to have a centralised health service research system alongside a long-established funder of biomedical research (the Medical Research Council) helps us interpret the significance of the English health research experience for other countries and helps English policy-makers better understand their present options.

Learning lessons from the features of the English health research system calls for an understanding of the processes which shaped it. Firstly, the publicly funded, nationally organised character of healthcare promoted government interest in evidence-based medicine, made research prioritisation simpler and helped promote the implementation of findings. Secondly, the essential role of leadership by a group who valued research for its health impact ensured that new management methods (such as metrics and competitive tendering) were harnessed to patient benefit, rather than as an end in themselves. A policy window of government willingness to invest in R&D for wider economic goals and buoyant funding of the health system were also effectively exploited.

## Background

In 2006, a National Institute for Health Research (NIHR) was established as part of a group of radical initiatives in United Kingdom research organisations. NIHR, with a 2015/2016 budget of £1037 million, is a virtual organisation within a government department, the Department of Health and Social Care (DHSC), responsible for the National Health Service (NHS)’s own research and its support for studies commissioned by others. (Until 2018 the Department was known as the Department of Health (DoH).) NIHR is the first body to manage this activity as a single system integrating clinical research, research capacity-building and research delivery by the health service — a reform that unlocked significant increases in government funding [1]. NIHR is by far the largest funder of clinical research in the United Kingdom. Examples of NIHR’s wide-ranging impact include clinical gains, such as the use of tranexamic acid to reduce serious bleeding and death after severe trauma, policy gains, such as its contribution (discussed later) to the work of the United Kingdom’s National Institute for Health and Care Excellence (NICE), and capacity development such as the establishment of the research leadership development programme [2]. This article is about England, the largest of the four countries of the United Kingdom; parallel developments took place in Scotland, Wales and Northern Ireland. (The expression ‘Britain’, used by two interviewees, often means the United Kingdom, though technically it excludes Northern Ireland.)

This history is of interest to an international audience because many of the challenges of health research are experienced worldwide [3]. An account of the English experience may illuminate how the publicly funded, nationally administered character of the English healthcare system has affected research and development (R&D). While Walter Holland compared health research in the United Kingdom and the United States of America, few other international comparisons exist — the present study, while not comparative, allows an international readership to understand the English experience better [4]. There has been very little study of the contemporary (post-1990) history of health research; Shergold and Grant’s review of health research in the United Kingdom since 1911 summarises the most relevant literature [5]. Our work covers the NIHR era in greater detail.

Our goal was a better understanding of ‘how we got here’ as an aid to current policy-making. To historicise a policy can help reframe debates, test assumptions, suggest alternative courses and help policy-makers understand “*the way in which their policies will mix into the flow of a society’s history*” [6, 7]. An explicitly historical methodology can supply otherwise unavailable insights for contemporary policy development because participants are more candid about past activities, seeing engagement via interviews as non-threatening. The establishment of the NIHR has been discussed by scholars of health research, but the present study is the first historical analysis, interviewing participants to gain insight into their motivation [5﻿, 8].

## Methods

All interviews were conducted by PA, joined on three occasions by SS. Both are historians and have undertaken the Oral History Society’s training in interviewing. Prior to the project, neither interviewer had had contact with the great majority of the interviewees. Thirty-eight interviews were recorded and transcribed, with participants being a mix of NIHR directors and other staff as well as senior figures from government and the world of research, granting us exceptional access to decision-makers. Sampling was purposive, with interviewees selected for our perception of their impact on policy-making. We took care to include interviewees who were involved behind the scenes or at more junior levels, rather than focusing exclusively on those who have been most visible and vocal.

We also searched and correlated primary and secondary historical sources such as policy documents and existing analyses with the interviews. Each interviewee received a tailored set of questions and points for discussion; interviews were semi-structured and ranged beyond these subjects when interviewees raised other material of interest. Oral history studies adopt a humanities approach rather than following a social science one such as grounded theory. Subjects were interviewed at locations they found most convenient, either their homes, workplaces or a neutral central London location (the Wellcome Library). Three interviews had to be conducted by telephone. There were no repeat interviews. Audio recording and transcription were used. All participants were also invited to an audio-recorded ‘witness seminar’ in February 2018 at the University’s London campus, which 22 attended; its transcript provided a further resource for the project [9]. Witness seminars add nuance and debate to the recollections of individuals, drawing out greater insight into the motivations behind past events.

Ethical approval was given by the University of Liverpool Committee on Research Ethics. Participants could choose to be quoted without prior consent, to approve quotations used or not to be quoted. Policy interviewing calls for an alertness to the interviewee’s agenda and power imbalances between interviewer and interviewee, and demands the maintenance of a critical distance; Berridge discusses these and other methodological issues about the validity of findings and how to avoid some of the pitfalls of policy interviewing [10].

## Results

The article discusses how NHS R&D interacted with four parts of its surroundings, namely evidence-based medicine (EBM), developments in public sector management, industrial policy and change in the NHS. These emerged from the interviews as ‘currents within the stream’ whose presence was essential to the eventual outcome. Research leaders had to navigate these currents: the successes of 2005–2006 described here were the result of their awareness of the developing opportunity, allied to a willingness to shape events. We examine each current in turn, before providing an explanatory synthesis. Table 1 provides a chronology.

**Table 1:** Chronology of NHS R&D

Before 1988	Research organised locally, plus government-sponsored policy research
1988	House of Lords report Priorities in Medical Research
1991	NHS R&D established
1993	Health Technology Assessment programme established
1993	Culyer Report on research funding commissioned
1997	Labour government elected, replacing Conservative (until 2010)
2000	Pharmaceutical Industry Competitiveness Task Force established
2000	National Cancer Research Network, a model for future networks
2003	Biosciences Innovation and Growth Team report
2004	Announcement of major increases in health R&D funding
2004	Sally Davies appointed Director of NHS R&D
2005	Consultation on integrated R&D strategy Best Research for Best Health
2006	NIHR established
2006	Cooksey Report A Review of UK Health Research Funding

### Evidence-based medicine

There would not be an NHS R&D programme without the EBM movement [11]. Drawing its inspiration from the work of the British clinical researcher Archie Cochrane as well as of David Sackett and Brian Haynes at McMaster University, Canada, EBM influenced clinical academics such as Iain Chalmers in the United Kingdom (Director, United Kingdom Cochrane Centre, 1992–2002; Co-ordinator, James Lind Initiative, since 2003) and then a wider circle of opinion formers [12]. By drawing attention to “*the problem of applying biomedical knowledge to the problems of live patients*”, EBM opened up a new angle in a long-running debate about whether health research was most productive when led by researcher interest or a ‘customer’s’ assessment of need [5, 13]. It enlarged both the field of enquiry for health research and the skills and methods that were admissible, though not without generating controversy [14].

Timo Bolt argues that EBM did not remain solely a critical thinking tool for individual physicians — its guidelines came to be used too by governments and other authorities [15]. The United Kingdom provided a good example of this by the end of the 1980s. As medical sociologist David Armstrong (Chair of the Medical Research Council (MRC) Health Services and Public Health Board 2004–2007, and later Programme Director, Research for Patient Benefit, NIHR) told us,

‘*in a way, the* [English] *individuals and organisations … were all part of this more general influence …, evidence-based medicine, the move towards deliverables and managerialism, impact … any organisation that got onto that bandwagon was going to succeed*.” (David Armstrong, interview 2 May 2017)

In the United Kingdom, government enthusiasm for EBM became clear with moves from the late 1980s to set up a Health Technology Assessment (HTA) programme. This coincided with a seminal House of Lords Select Committee Report entitled Priorities in Medical Research [16]. Our participants saw this Report as a key moment:

“*It was about the fact that if we invested so much in health services then we should have an intelligent commissioning arm that made sure we had research to address our needs, and arising out of that we got the NHS R&D programme*.” (Sally Davies, Witness seminar 28 February 2018) [9]

Michael Peckham, appointed as the programme’s first director in 1991, funded systematic reviews, the United Kingdom Cochrane Centre and HTA, all central to the later NIHR mix [17]. This HTA programme differed from those in many other countries in having free rein to generate data, not just to synthesise data created by others — hence, it could set its own priorities for commissioning very large and expensive pieces of data generation (trials). Tom Walley, the HTA’s Director from 2004 to 2015, noted: “*People at the time often referred to the HTA Programme as the ‘jewel in the crown’ of the R&D Programme, basically because it was the only jewel*” (Tom Walley, interview 23 February 2017).

The United Kingdom was a good place for HTA to thrive in the 1980s, partly due to government efforts to control rising healthcare costs [18]. The United Kingdom’s policy-making architecture, in particular central government’s direct responsibility and political accountability for funding the NHS as a centralised healthcare system, ensured that governmental concerns about rising healthcare costs would be especially strong [19]. In establishing the HTA programme, Peckham thoroughly embraced a health economics perspective — that necessarily finite resources ought to be spent on the things that improved health the most.

The United Kingdom’s political environment was to become more favourable to EBM. The Margaret Thatcher Conservative governments of the 1980s had experimented with introducing the New Public Management into the NHS — the Griffiths Report of 1983 on NHS management being a key example [20]. In 1988, Thatcher announced that the NHS was to be reformed. There was to be an institutional separation of the commissioners and the providers of healthcare through the creation of an internal market in which, in principle, competition would contain costs and drive quality improvements, in part by using evidence [21]. Implementation took place progressively from April 1991. As Peckham saw, “*the reforms in a way began to introduce the notion of, ‘should we buy that rather than that?’ which was knowing about cost-effectiveness and evidence*” (Michael Peckham, interview 23 August 2017).

The influence of EBM on NHS R&D was two-fold. Firstly, it influenced what was commissioned; the growing influence of the EBM movement shifted the research agenda from the ‘laboratory bench’ towards the ‘bedside’ end of the spectrum, pushing it to seek better care by studying the ‘Scientific Basis of Health Services’ (the title of a 1995 conference mounted by the DoH’s Research and Development Directorate (RDD), and of the published proceedings) [22]. This trend can be seen in published funding figures (though these have their technical problems), where the largest government and charitable funders spent 69% of their funds on basic health science in 2004/2005 and 15% on the most ‘applied’ research; in 2014, the corresponding figures were 52% and 20% [23, 24]. (Here, ‘basic science’ refers to the categories ‘underpinning’ and ‘aetiology’; ‘most applied’ refers to ‘treatment evaluation’, ‘disease management’ and ‘health services’.)

Secondly, by emphasising the place of evidence about healthcare in the research agenda, the EBM movement bolstered the view that the NHS itself should commission research. As the House of Lords report argued, if research about effective healthcare is needed, then the health system is the group best placed to prioritise and commission it [16]. The NIHR’s needs-led selection of research priorities illustrates an EBM approach. This was an important motivation for Sally Davies, who, as the DoH Director of R&D from 2005 to 2011, led the establishment of the NIHR. (Davies was Director of R&D, NHS London North and then London Regional Offices, 1997–2003; Deputy Director of R&D, 2003–2005, Director of R&D, 2005–2011, and Chief Medical Officer, 2011–2019.) In the words of George Binney of Ashridge Management College, one of her management mentors:

“*back in 2004/05, Sally was clear, the themes … were the result of her experience … that passion, to be a part of that movement, the need for a much more evidence-based care … She was at the heart, and believed profoundly, in that shift: the need for decent evidence*.” (George Binney, interview 14 July 2017)

### Developments in public sector management

If EBM prepared the scientific ground for the NIHR, the ‘New Public Management’ can be said to have prepared the managerial and political space. In the United Kingdom, from the 1980s, the marks of the New Public Management, which set out to use private sector templates to reform public sector management, included privatisation, managerialization, marketization, top-down reform, the search for value for money, the use of performance indicators and emphasis on the management of change [25]. All of these elements are visible in the changing ways in which NHS R&D were managed. Cutler and Waine describe how, in power after 1997, New Labour (in contrast to old Labour’s rejection of managerialism and markets) took much of the New Public Management on board in a ‘reformed managerialism’, which attempted a more rounded approach to performance measurement and tried to balance competition with ‘partnership’ [26]. Christopher Hood has noted the particularly British (and, above all, English) growth of interest in using targets and rankings, ascribing this largely to the exceptional scale and centralisation of institutions such as the NHS, one of the themes of the present article [27].

Management consultants were an important vector for the New Public Management, in R&D as elsewhere. Sally Davies recalls that ‘*a variety of people played key roles* [in writing the main strategy paper Best Research for Best Health], *but I would argue that Jonathan Grant and the organisation he worked with, RAND Europe, played key roles*” (Sally Davies, Witness seminar 28 February 2018) [9]. NHS R&D were using RAND Europe as consultants in this period on various projects. (RAND Europe is a not-for-profit management consultancy organisation whose mission is to help improve policy and decision-making through research. Grant worked there on 2002–2012, becoming President in 2006).

In R&D, there was a new stress on managing research programmes, copying the HTA programme model, with a balance of directly commissioned and researcher-led projects. This contrasts with Stephen Davies’ description of an earlier era (pre-1967) of ‘enlightened patronage’ in which project ideas came mainly from the “*informal team*”, working on a discretionary basis with limited oversight and growing budgets, in a system in which “*personal connections and networks were pivotal*” [13]. To Russell Hamilton, the Deputy Director of R&D from 2003 to 2011, who brought the new emphasis on management to its most highly developed form, the attraction of an innovation such as the Cancer Research Network (established in 2000), was that it was:

“*a managed clinical research network, and the people in the network most loved the fact that it was clinical and it was a network, the researchers most loved the fact that it was about research, and I most loved the fact that it was managed.*” (Russell Hamilton, interview 31 May 2017)

Another feature of the new approach that heralded the NIHR was a greater emphasis on, to quote the NIHR’s launch document Best Research for Best Health, “*transparency, fairness and contestability*” [28]. Everyone would know why funding had been granted to one piece of research over another; researchers in each geographical area would have the same chance, and there was a new focus on evaluation, championed by Russell Hamilton. As Nick Black (Professor of Health Services Research at London School of Hygiene and Tropical Medicine since 1995) noted:

“*Russell was important in terms of the culture, that was the switch. I … had one or two run-ins with Russell, because I found his approach a bit too, red in tooth and claw, … always he said, more or less, ‘what’s the cost-effectiveness of doing that?’ … he was pretty challenging on, ‘where’s the evidence that that’s going to be worth doing?’*” (Nick Black, interview 25 January 2018)

The ground for this emphasis on ‘payback’ had been prepared in the 1990s, when the NHS HTA programme had first commissioned the development of payback analysis tools [29, 30]. One such analysis later found that the HTA programme had “*considerable impact in terms of knowledge generation, as well as a perceived impact on policy and to some extent on practice*” [31].

Contracting out the management of research programmes to co-ordinating centres sharpened the specification of services and outputs, and the monitoring of delivery, an example of Hood’s general observation that “[w]*hat seems to be different about the recent past is the greater top-level political salience given to measured performance targets*” [27]. Enrolment in clinical trials was a prominent example.

The spread of a managerial desire to measure and see rapid results affected what research was done as well as how it was managed. The emphasis David Armstrong noted on ‘deliverables and managerialism’ led, in his view, to more interest in trials. He gave the example of the Cancer Research Campaign, which:

“*had realised that this was where the agenda was … all the medical research charities* [were] *moving in that direction …* [if] *you fund a trial, you can say to all your members, to all the people rattling the money boxes, … ‘look, this is what we’ve done’.*” (David Armstrong, interview 2 May 2017)

Elsewhere in his interview, Armstrong reiterated that trials were “*good science to fund … , especially in a managerial sense*”. The emphasis on payback has encouraged attention to the dissemination of results in accessible forms to people who will use them, for example, through the NIHR Dissemination Centre’s Signals, Highlights and Themed Reviews [32].

The influence of the New Public Management in Whitehall from the 1980s created the conditions in which top-down implementation of a reform strategy, requiring management, transparency, measurement and payback, could thrive. NHS R&D leaders from John Pattison (Director of R&D, DoH, 1999–2004) onwards embraced the opportunities those conditions offered, not to pursue a management agenda for its own sake but to secure new research goals — particularly, higher quality, from commissioning to dissemination — which called for more control over research processes than hitherto.

Indeed, the contributions of individuals were critical to the changes of 2005–2006 that created the NIHR. Pattison was significant for building Ministerial belief in the NHS R&D programme — an essential platform on which his successors, Sally Davies and her deputy Russell Hamilton, could build. The extra feature which Davies and Hamilton brought was the determination to bring about an integrated research system, even in the face of vested interests keen to retain control of their own research funds. Their approach combined a grip of detail with a dedication to winning over the researchers.

### Industrial policy

A new industrial policy arrived with the New Labour government in May 1997. In the words of Treasury official Paul Devenish:

“*The Chancellor* [of the Exchequer, Gordon Brown] *had an agenda … post-neoclassical endogenous growth theory, the driver of growth in the economy was productivity, … productivity is obviously linked to R&D investment, and because the pharma industry … was doing 25% of United Kingdom private sector R&D at the time, … that was seen by the Chancellor as key to his productivity agenda.*” (Paul Devenish, interview 3 May 2017)

Prime Minister Tony Blair took the same view [33]. This point of view was a departure from the thinking of the previous administration, as John Kingman, Director of the Treasury’s Enterprise and Growth Unit (2003–2006) recalled:

“*The Treasury’s positive interest in the case for investing in science, R&D and innovation, really took off under Gordon Brown.*” (John Kingman, interview 6 July 2017)

Kingman played a key role in advancing this outlook through his networks:

“*it was part of our job to be out and about talking to, certainly people in the life sciences, venture capitalists, … big pharma, we would be talking to Vice-Chancellors, that was part of the job of that part of the Treasury, to have those relationships and to know what people thought. … John Bell and David Cooksey were the obvious people.*” (Bell is Regius Professor of Medicine at the University of Oxford, Cooksey a venture capitalist whose role in the Bioscience and Innovation Growth Taskforce and elsewhere is discussed below.) (John Kingman, interview 6 July 2017)

Hugh Taylor, DoH Permanent Secretary 2006–2010, recalls Sally Davies’ regular meetings with Kingman, Cooksey and others: “*they were all about focussing the government-level interest in health sciences and its relationship with R&D …, a high mark of government cross-Departmental emphasis on this*” (Hugh Taylor, interview 12 June 2017).

The new thinking on productivity was known in the health sector as ‘health and wealth’. In 1997, many health researchers and officials were, in Russell Hamilton’s words, “*very, very, very uncomfortable about the ‘wealth’ bit*” (Russell Hamilton, interview 31 May 2017). They suspected that companies’ trials tended to favour their own products, and in any case compared them with placebo to show efficacy, when the NHS interest was as much in efficiency — whether the new drug worked better than the existing one.

One of the earliest examples of a new enthusiasm to smooth the way for commercial R&D was the establishment in March 2000 of the Pharmaceutical Industry Competitiveness Task Force (PICTF), following a meeting between Blair and the CEOs of AstraZeneca, Glaxo Wellcome and SmithKline Beecham [33]. As we will see, overcoming impediments to commercial clinical research in the NHS was not their most pressing concern (John Kingman, interview 6 July 2017). Philip Hunt, a junior Minister in DoH, was particularly instrumental in seeing that healthcare industries, including pharmaceuticals, came to be seen as legitimate stakeholders in DoH policies.

John Pattison, the Director of NHS R&D from 1999 to 2004, brought in Sally Davies to assist in PICTF’s high-level working group on clinical research, which he co-chaired with Vincent Lawton, the Managing Director of Merck, Sharpe and Dohme [34]. This was no inevitable choice — from 1997 to 2003 Davies was one among eight Regional Directors of R&D. She had, however, already demonstrated her interest in the connections between the NHS and industry. Around 2000, she had produced a report on the economic impact of health on London, “*looking*”, in the words of Nigel Crisp, then her regional Director, “*at health as an economic factor*” (Nigel Crisp, interview 11 September 2017). Davies recalls, during her London region period, being introduced to George Poste, then in a top management role at SmithKline, who, in her recollection, “*was very well networked and he came and mentored me and gave me lots of good advice*” (Sally Davies, interview 6 June 2017).

During 2000, Davies spent a month on secondment with GlaxoSmithKline R&D arranged by Dr Tadaka Yamada, its head of R&D. In the United Kingdom civil service of this period, such secondments were very unusual, and for her to seek one out demonstrates an unusual dedication to understanding how the interactions between industry and her official responsibilities worked. Pattison recognised Davies’ suitability by making her the Portfolio Director for industry — a cross-cutting brief covering all NHS R&D.

The remit of the Clinical Research working group was carefully drafted to “*review … the opportunities and costs associated with the clinical research infrastructure in the NHS as a base for research by pharmaceutical companies, in tandem with promoting and supporting R&D of value to patients and the health service*” [34]. Here, in a nutshell, was the ‘health and wealth’ argument for making the NHS a more congenial place for commercial clinical research.

The new part of ‘health and wealth’ thinking, which was later significant in the emergence of NIHR, was the proposition that the more commercial clinical trials took place in the NHS, and the more NHS patients were enrolled in commercial clinical trials, the better; indeed, NIHR established benchmarks for enrolment numbers [35]. The ‘wealth’ benefits were mainly greater R&D investment in the United Kingdom; the ‘health’ benefits were “*the development of beneficial treatments for NHS patients … keeping the NHS at the forefront of modern treatments and research*” [33]. For Hamilton, it was important to NHS R&D leaders that the treatments were beneficial ones: “*not just ‘open our doors to anything’ … we have systems in place to make sure that the industry studies that are supported … meet quality thresholds, so that we can be confident that the research we’re supporting is of benefit to patients*” (Russell Hamilton, interview 31 May 2017). Industry wanted the NHS to make clinical trials quicker and cheaper, and the working group found convincing arguments that patients, as well as pharmaceutical companies, would benefit, attracting more commercial research to the NHS.

Our interviewees spelt these arguments out further. Davies drew attention to research data showing that “*outcomes* [for patients] *are better, even if you’re not in a trial, in a research-active environment. And then there’s the data that patients like being in trials*”. (Sally Davies interview, 6 June 2017). Hamilton added that:

“*Sometimes … what patients want is access to the latest medicines, and one of the ways of getting access to the latest medicines, and your clinicians and NHS geared up to deliver the latest medicines, is by doing industry research, and, at least when we first started …, there was a perception that in clinical trials, the standard that was required of an organisation … doing pharmaceutical industry research was higher than that for public sector funded research: you needed better record-keeping, better protocols, more inspection*.” (Russell Hamilton, interview 31 May 2017)

In the words of her colleague Louise Wood (Director in R&D, 2005–2016, Director of Science, Research and Evidence, 2016 to present, DoH/DHSC), “*Sally had … really taken to heart what she had been exposed to and learned through that*” (Louise Wood, interview 6 June 2017).

After PICTF’s ‘Final Report’ in March 2001, a Ministerial Industry Strategy Group held DoH to account for delivery — good intentions were not enough [36]. In 2002, Lords (David) Sainsbury and (Philip) Hunt, Ministers respectively at the then Department of Trade and Industry and the DoH, convened the Biosciences Innovation and Growth Team (BIGT). This sprang from similar origins to PICTF — Ministers met the Bio-Industry Association and announced a shared goal “*that the UK should maintain and develop its position as a global leader in the biosciences*” [37, 38].

Ministers appointed the venture capitalist Sir David Cooksey as BIGT’s chairman. Cooksey has played an important role in the interaction between industrial policy and health R&D. He told us that “*I passionately believe … the thing that has moved our economic wellbeing forward has been scientific discovery and the application of it*” (David Cooksey, interview 6 June 2017). Cooksey’s energy, success as an investor and preoccupation with the international competitiveness of the United Kingdom’s life sciences industries have made him a valued contact for Ministers, Conservative and Labour. He recalls that “*Gordon* [Brown] *really got to grips* [in 1997–2000] *that we could really upgrade Britain’s performance in science very considerably, it has worked very well*”. Cooksey’s road to the heart of industrial policy included an appointment as a governor of the Wellcome Trust, the United Kingdom’s largest charitable health research funder, from 1995 to 2000, where he influenced its policy thinking substantially.

Cooksey was also an influential supporter of Davies’ goals for NHS R&D, seeing her as one of the “*people … who actually saw this thing of turning good technology into health and wealth*”. We have already seen how John Kingman at the Treasury was another such supporter. More generally, a whole industrial policy network saw Davies as a valuable asset, likely to deliver more of their goals for NHS R&D than anyone else would. Hugh Taylor recalls:

“[Davies] *came in for quite a lot of heavy-duty criticism … from some elements of the science community … she had very strong protection of course, she was well-connected in the Treasury and No. 10* [The Prime Minister’s Office]. *In terms of Whitehall politics Sally was always well-guarded … people in industry saw that she was a shaper of stuff in a way that was quite significant.*” (Hugh Taylor, interview 12 June 2017)

The 2003 BIGT report was candidly entitled Bioscience 2015: Improving National Health, Increasing National Wealth [39]. Alongside its more ‘industrial’ recommendations, BIGT recommended increased spending on NHS R&D, and better collaboration between the NHS and industry. The Government accepted all BIGT’s main proposals, and the DoH set up the Research for Patient Benefit Working Party to develop a suitable response to the BIGT report, and to John Bell’s contemporaneous report for the Academy of Medical Sciences, Strengthening Clinical Research [40, 41]. Cooksey’s own verdict was that BIGT “*produced Biosciences 2015 and produced NIHR*” (David Cooksey, interview 6 June 2017) — an understandable exaggeration, given his own role.

John Pattison was very clear about the centrality of industrial goals in 2003: “*Ministers wanted something to follow on from* [PICTF]*, putting it crudely, in helping the pharmaceutical industry stay in the United Kingdom. We could not have a working party called Helping the Pharmaceutical Industry Stay in the UK*, *so we called it Research for Patient Benefit Working Party*” (John Pattison, Witness seminar 28 February 2018) [9, 42].

A network of research nurses to support cancer clinical trials had been shown to boost recruitment to trials substantially. David Kerr, a leading cancer researcher, and John Bell therefore called on John Pattison in autumn 2003 to encourage him to copy the clinical research network model from cancer into other fields [35]. NHS R&D staff had greatly increased patient enrolment rates in treatment trials for cancer between 2000 and 2004 [34]. There has been particular emphasis on trials in the field of cancer [43]. Pattison used this success when asking Treasury for funds in March 2004: “*the aim* [with cancer research] *was to double the number of patients in clinical trials … the target was achieved a year ahead of schedule … one indication that Britain could be the best place for R&D and innovation in the world. We need to replicate this success in other areas of medicine*” [35, 44]. The result was a greater orientation — in presentation, if no more — towards clinical trials in NHS R&D than previously.

Consultation in July 2005 on the DoH’s ambitious Best Research for Best Health — Sally Davies’ blueprint for NIHR — led to renewed tensions between the MRC, which distributes government funding mainly for fundamental biomedical research, and the DoH [45]. Earlier relations between the two are described by Shergold and Grant [5]. In late 2005, the Treasury gave Cooksey the task of “*advis*[ing] *on the best design and institutional arrangements for public funding of health research in the UK*” [46]. Cooksey had convinced Treasury officials that the existing arrangement between the two bodies on who funded what was not adequately supporting translational research. (‘Translational’ research means ‘*translating ideas from basic … research into the development of new products and … treatment*[s] *… ; and implementing* [these] *into clinical practice*” (John Kingman, interview 6 July 2017). Much public attention focused during the review on possible organisational changes such as routing all research funding for medicine and health through the MRC (which had many influential academic supporters), or all clinical research funding through NIHR, or even merging the two bodies [47, 48].

The genesis of the Cooksey review suggests that the Treasury did not expect it to have the high profile which it has had in its afterlife. Kingman recalls: “*I think we were sitting in the Treasury thinking ‘if we’re to achieve everything we want to achieve in the life sciences, having the MRC and the Department of Health at war obviously is not part of the solution’*” (John Kingman, interview 6 July 2017). In the recollection of Paul Devenish, who was the review’s secretary: “*David* [Cooksey] *kept coming in and lobbying and John* [Kingman] *was sort of saying ‘we have to do something about* [it]*, well what can we do?’ I said ‘… David’s so keen on this, why don’t we get him to run a review?’*” (Paul Devenish, interview 3 May 2017).

Some interviewees made much of the astringent relationship between Davies at the DoH and Colin Blakemore, the Chief Executive of the MRC, between 2004 and 2007. This was real — but there is no reason to think it made much difference to the structures and processes that emerged. Cooksey deserves credit for avoiding the pitfall of structural change as an easy option. Instead, his 2006 recommendations about research organisation, largely adopted, kept the MRC’s structure and that of the NIHR (which the government, implementing Best Research for Best Health, had opened in April 2006) mainly unchanged.

A new Office for the Strategic Co-ordination of Health Research was charged with setting strategy, distributing the research budget, monitoring results and preparing bids [46]. Under John Bell’s chairmanship, it proved to be the stage on which NIHR and the MRC demonstrated increasingly close working relationships, and secured rising funding. As he put it: “*by swimming together … in roughly … the same direction, you would get more, and the government would get more, and the research community would get more*” (John Bell, interview 13 June 2017). It was agreed that NIHR would take responsibility for clinical research and MRC for biomedical science and preclinical studies. When BIGT reported again in 2009, its comments on NHS R&D were overwhelmingly positive, noting that “*early signs are that* [NIHR Clinical Research] *Networks are having a significant impact on* [clinical trial] *delivery*” [49].

Was the enthusiasm among health R&D leaders for the NHS to facilitate commercial R&D instrumental — selling R&D to Ministers and the Treasury by promising that investment would serve their new industrial policy goals — or did it stem from a conviction of the policy’s intrinsic merit? Key research leaders whom we interviewed have argued all along that these policies were good for ‘health’ and ‘wealth’ — a stance maintained in our interviews. For example, Davies is clear about both the intrinsic merits of ‘health and wealth’ and its instrumental value:

“*It was me that pushed it and me that has always banged on about industry. … what I’m good at* [is] *sniffing and saying, ‘so that’s where the wind’s going, how do I put my sail up so I think it will sail me where I want to go?’*” (Sally Davies, interview 6 June 2017)

The intrinsic merits of ‘health and wealth’ are perhaps best expressed by Wood:

“*Sally, … all of us, … saw intrinsic merits in it. … if we as a country were unable to bring in resource, ultimately who’s going to be paying our pensions, who’s going to be funding the NHS? … we genuinely believed there was an area of shared interest between industry and the NHS.*” (Louise Wood, interview 6 June 2017)

Hamilton added that tailoring arguments to different audiences was no more than intelligent advocacy. He emphasised too that supporting industry was one among many goals, a point borne out by examination of Best Research for Best Health and by the balance of NIHR’s activities:

“*One* — ***one*** — *of the important aspects of NIHR, is to improve the wealth of the country, to support industry, … When we’re focussing on that, we absolutely say how many industry trials we’re supporting, what the inward investment … benefit to society is, how many jobs have been created … , all those hard economic indicators — when we’re trying to demonstrate that. When we’re trying to demonstrate something else,* [say] *the reduced mortality from cancer, then we focus on that, pure health thing*.” (emphasis in the spoken original) (Russell Hamilton, interview 31 May 2017)

As historians, we are inclined to accept the argument in this testimony. While it is possible that these individuals were talking about ‘health and wealth’ purely for instrumental reasons (securing more government funds and more political buy-in), the weight of the evidence suggests to us that our witnesses also believed the new stress on the NHS facilitating commercial R&D would benefit patients — and that they were largely correct.

‘Health and wealth’ was much more the initiative of New Labour politicians than the result of lobbying by industry. While there is copious evidence for support from Ministers, civil servants and a few external advisers (notably Cooksey), there is little evidence that pharmaceutical industry figures lobbied government to invest more in health research (they were more interested in pressing for faster NHS uptake of new therapies). In the United Kingdom, the framework for drug companies’ interaction with government is a central scheme which regulated the profit that they could achieve on sales to the NHS. Our project approached pharmaceutical industry leaders from the period but only one, Richard Barker (Director General, Association of the British Pharmaceutical Industry, 2004–2011), agreed to an interview. Barker stressed the industry’s interest in ensuring trials continued to take place in the United Kingdom, for example, by simplifying the approvals process (Richard Barker, interview 12 June 2017).

### Change in the NHS

The most rapid development in NHS R&D coincided with the period of most rapid change in the NHS. We have seen how the DoH separated NHS R&D from the rest of its in-house research programme in 1991. This led to an exciting period of innovation and development; however, this rapid change was not taking place in a vacuum.

Thatcher’s 1991 purchaser–provider split improved the status of HTA and EBM, as we discuss above. The internal market reforms shaped NHS research in further key ways. Since the need to negotiate contracts placed the spotlight on providers’ costs, NHS provider managers began to focus on the costs and benefits of research taking place in their institutions. This was a substantial percentage of the running costs of a teaching hospital, making its prices uncompetitive with those of hospitals, which had less research. The political consequences of undermining the financial viability of prestigious London institutions such as St Bartholomew’s Hospital could not be countenanced. Marc Taylor (head of DoH R&D policy, 1999–2005) recalls: “*Every policy change that happens in the NHS is tested against whether Barts will go bust, of course, but we seemed to be particularly in the spotlight, being accused of making Barts go bust*” (Marc Taylor, interview 21 August 2017).

NHS reform therefore drove an identification of research costs. The DoH moved rapidly to protect its internal market reforms from the threat posed by research. Senior NHS finance officials were pressing for a review of existing funding support for R&D, to make research funding transparent and accountable — two watchwords of the internal market enterprise. This aligned with Peckham’s wish to protect NHS research from the effects of internal market competition, such as pressure on doctors for ever greater clinical activity. Political pressure for this mounted until, as Keith Peters, Regius Professor of Medicine at Cambridge, noted:

“*Partly stimulated by a letter which Max Perutz, the head of the* [MRC Laboratory for Molecular Biology], *wrote to* [Prime Minister] *John Major, which I had a hand in drafting, there started to be some serious debates at a higher level about the problem of teaching and research in the reformed health service.*” (Keith Peters, interview 6 July 2017)

In response, the government announced, in November 1993, the establishment of a Research and Development Task Force chaired by Anthony Culyer, a health economist from the University of York. The Task Force’s recommendations would transform the NHS’ R&D funding mechanisms and directly lay the foundations for Sally Davies’ more radical reforms [50]. Existing flows of money for research would be laid bare, and gradually redirected where they could buy most research impact, in a kind of internal market for clinical research mirroring the larger internal market for healthcare. According to Marc Taylor: “*this review, although it looked as though it was much about finance … it anchors back into all of the other elements of the policy that Peckham brought in*” (Marc Taylor, interview 21 August 2017). It took the radicalism of Sally Davies’ approach in 2004–2006, however, to make a reality of the goal of centralising decisions about the allocation and spending of the money Culyer had identified. Against entrenched opposition, most had thought this would not be achievable.

The 1997 Labour election victory brought a change of political mood. In Jonathan Grant’s words:

“*There was just, optimism, which was probably not just about medical research, but about everything, a sort of can-do type attitude … it felt an exciting and opportunistic time where things could occur. … that … ma*[d]*e the mood music sort of positive.*” (Jonathan Grant, interview 31 October 2017)

Philip Hunt, the Minister responsible for health research from 1999 to 2003, describes the enthusiasm for research felt by incoming Ministers:

“*I think it was a mood, about this nation reinventing itself, that fitted very much with the Tony Blair and Brown philosophy, alongside what we were doing for patients, and you put the two together and you get that feel of something dynamic and exciting*.” (Philip Hunt, interview 27 February 2018)

Hunt noted that Alan Milburn, Secretary of State for Health from 1998 to 2003, shared this outlook:

“*Milburn was up for this. You cannot ignore, if you’ve got a Secretary of State who’s interested it makes life so much easier. … in terms of decision-making and strategic intent I think he was good, and he was up for this agenda*.” (John Kingman, interview 6 July 2017)

New Labour slogans of this period, including ‘investment for reform’ and ‘what works’, suggested support for research to help the NHS identify what ‘worked’ [19]. In 2000, Blair announced major growth in NHS funding (John Kingman, interview 6 July 2017). This soon improved allocations for NHS R&D. After that, government policy actively called for more and better research, and enabled it (starting with cancer). Labour’s creation of the National Institute for Clinical Excellence (NICE — now the National Institute for Health and Care Excellence) in 1999, charged with ensuring that NHS patients had access to the most clinically and cost-effective treatments, is a good example of policy initiatives that favoured research — at one point, NICE was said to be the customer for half of all the HTA programme’s output.

The NHS has always experienced stop-go funding; R&D budgets have not been immune to its effects, and money allocated to R&D was diverted to meet short-term service pressures in 1998 and 2001 [5]. While Michael Peckham had been allowed to talk publicly about the aspiration of spending 1.5% of the NHS budget on R&D, the reality of R&D funding remained very tight until after 2000. Rapid expansion followed, especially in the 2004 Comprehensive Spending Review. John Pattison

“*was absolutely astonished at that settlement, completely surprised. It just felt as if Gordon Brown was saying, ‘I have taken care of the money, now you show us what you can do’*” (John Pattison, Witness seminar 28 February 2018) [9].

While funding became tighter after the banking crisis of 2008, this positive environment for research continued (a testimony to the convincing arguments being made by 2008 about its value for money). It is plausible that this exact succession of two different stimuli was ideal for fostering good NHS R&D in large quantities — adversity, but not too much, to seed the pearl, then prosperity to grow it.

Intimately linked with funding was the fate of the NHS’ regional organisation, which had been an essential player in research. Peckham had developed the regional R&D structure assiduously, appointing respected research leaders, such as George Alberti and Alastair Breckenridge, to key regional posts but, in times of financial stringency, Ministers cut regional tiers of administration. The downgrading and eventual closure of regional structures began in 1996 and was complete by 2003, along with the loss of the NHS’ capacity to manage research above the level of individual providers, necessitating the centralisation of research funding [51].

This constrained Davies’ policy choices, though the timing was fortunate for her — regional staff had been essential to the successful identification of existing research spending in the 1990s (Chris Henshall (Assistant Secretary then Deputy Director of R&D, Department of Health, 1991–2001), Witness seminar 28 February 2018). Thereafter, a single national organisation was more responsive than a regional structure would have been to Davies’ vision for the delivery of NHS R&D.

## Discussion

The interviews and witness seminar provide a complementary range of perspectives on how the NIHR came into being and why it took the form it did. This oral history approach adds to our understanding of current challenges in health research such as funding, sustainability and scope. Describing the period after 2000, John Pattison recalls:

“*There was an issue about cancer. And then all the other stars seemed to align themselves, when you think about it. Because cancer was as good an example as any that you might find to pilot, as it were, all sorts of things that would be of benefit to the pharmaceutical industry — let’s put them in rank order — patients, the National Health Service, the pharmaceutical industry, and the government itself.*” (John Pattison, interview 7 June 2017)

Pattison’s concern to list the stakeholders in the right order here suggests the sensitivity of the ‘health and wealth’ argument for research reform. Hugh Taylor adds:

“*Around 2004 … a combination of things* [came] *together. One was Sally* [Davies]*’s appointment. Clearly, she and Nigel* [Crisp, Chief Executive of the NHS] *had a good rapport …, and she came with some ideas, but also … there was then a mounting sense of interest in the science agenda across government. … And of course the overall budgetary position was easier. I guess more questions were being asked about, to put it bluntly, what the money was being spent on, where it was going.*” (Hugh Taylor, interview 12 June 2017)

What does this history have to say to audiences beyond the United Kingdom? A brief comparison with three other countries which have also taken an interest in EBM — the United States, Canada and the Netherlands — may be informative here. These countries have funded health research in somewhat different ways. In the United States, Holland noted the impressive depth and diversity of sources of research funding, including in his own field of epidemiological public health. Schools of public health, philanthropic foundations and the National Institutes of Health all contributed to this. His overall verdict was that fragmentation of health services among many payers meant that identifying priorities for public health research was harder. “*US research is more ‘democratic’ and ‘investigation’ rather than ‘problem’ driven. The lack of structure also implies that the translation of research findings into health or health services policies is far more problematic*” [4].

Part of this problem of translation, in the United States, has been a growing perception among some politicians that health services research was partisan, and favoured the Clinton and Obama healthcare reform agendas. This has created a fraught environment for engagement between researchers and policy-makers [11]. In contrast, in the United Kingdom, the ability of John Pattison and Sally Davies to boost the credibility of NHS R&D with Ministers paid dividends. DoH research commissioning rose from £97 m in 2004/2005 to £218 m in 2009/2010 and £286 m in 2014/2015 [52]. Sally Davies is clear that this is partly due to the sense of Ministerial ownership which flows from running the programme within the DoH. The concomitant risk of political intervention in commissioning has been mitigated by a calculation on Ministers’ part that — in a somewhat similar way to their arm’s-length management of service delivery — leaving decisions to NIHR avoids political argument about accountability (Sally Davies, Witness seminar 28 February 2018).

In both the United States and the United Kingdom, the producers of guidelines have found it most effective to involve healthcare funders and the health professions as well as patients and the public in co-production. In the United States, the National Guidelines Clearinghouse (established in 1998) and the Patient-Centered Outcomes Research Institute (established in 2010) have been significant examples of this [53].

Canada established the Canadian Institutes of Health Research (CIHR) in 2000, bringing together disparate elements of central funding, alongside provincial funding of health research. The CIHR, which has 13 virtual institutes for different research fields, has a mandate to promote multidisciplinary research and translation into patient benefit. England’s NIHR was a less organisationally radical step in the same direction. Both benefited from an early major injection of extra funding. A review of the CIHR suggested its impact had been highly positive, though identifying challenges in the co-ordination of different funders which resemble those seen in England (John Kingman, interview 6 July 2017). Canada also benefits from a Canadian Health Services Research Fund, funded by an initial endowment (which provides more independence from government than English equivalents). Its mission involves bringing together policy-makers and managers with health services researchers to foster the co-production of research, which is more likely to change practice [54]. The NIHR’s Service Delivery and Organisation programme, and its successor, the Health Services and Delivery Research programme, have a similar outlook on co-production.

In the Netherlands, the health research landscape is characterised by pluralism, with a range of public and independent bodies such as ZonMw (Netherlands Organisation for Health Research and Development), NIVEL (Netherlands Institute for Health Research) and RIVM (National Institute for Public Health and Environment). In 2001, the medical research part of NWO (National Organisation for Scientific Research) merged with the research organisation of the Ministry of Health to form ZonMw [55, 56]. This innovation is very similar to one of the options rejected in England by the Cooksey Review, and also resembles the CIHR. While each institutional context is unique, these experiences at least indicate worldwide interest in organising health research to emphasise the ‘problem’ rather than the ‘investigation’ focus. Another feature the Netherlands shares with the other countries discussed is innovation in forms of co-production in health research [57]. The Netherlands also has researchers and institutions with an active interest in examining research ‘payback’, a feature this paper has noted in England with NIHR [55].

This comparison with three similar countries suggests some messages for non-United Kingdom readers, at least in higher-income countries and perhaps more widely. Health research appears to achieve most where R&D leaders can establish their political credibility with Government rather than be viewed as outsiders — structures which give government ministers a sense of ownership (without sacrificing scientific independence) appear to help. Unsurprisingly, levels of government investment in health research are critical, but it also matters what strings are attached — slightly less with more freedom over how to spend it may be better than slightly more under tight political control. Research leaders’ ability to earn politicians’ trust may be key to achieving that. Two measures likely to contribute to political support are to place the greatest emphasis on ‘problem’ rather than ‘investigation’ research, and to devote attention to measuring and reporting research ‘payback’. Finally, research leaders find it valuable to secure the participation and buy-in of healthcare funders, health professions, patients and the public, not least in the development of treatment guidelines.

## Conclusions

Hanney et al. consider that the English reforms have been able to meet the needs of many stakeholders, and “*could usefully inform attempts elsewhere to develop health research systems that are similarly responsive*” [8]. We recall, though, that learning travels most effectively from one country to another when there is understanding of the varying context [58, 59]. Our research shows, by adding the historical dimension, that, for an international readership, four points are salient about the English experience.

First, there was an openness to applying the EBM approach at the level of services as well as clinical practice (though less so at the level of governance). The publicly funded, nationally organised character of the NHS gave an impetus to this, because EBM supported a national emphasis on using research to get better value for money. Second, the existence of a health service research organisation after 1991, with high-profile research leaders, meant that reform was steered and championed by people such as Pattison, Davies and Hamilton, who assessed health research for its patient benefits and not simply for organisational benefits such as efficiency. The application of ‘New Public Management’ techniques such as the use of metrics was the servant and not the master.

Third, the policy window of ca. 2000, with its specific policies on public R&D investment, allowed research leaders to harness ‘health and wealth’ arguments to lever extra government resources for health research. Governments are not always willing and able to respond in this way. The public-sector nature of English healthcare also meant that government had much stronger levers with which to use health research in industrial policy — measures to improve the research infrastructure for commercial clinical trials would have taken a less direct form in a health system of private providers.

Fourth, while English conditions heightened the impact of government research policy on the health system, they also heightened the effects of changes in the health sector on research policy — in particular, fluctuating service funding in the 1990s and 2000s as well as the 1991 separation of purchasers from providers. To summarise, the close links between England’s health system and health research system meant that, when the latter, under Sally Davies, wanted change, it rapidly reached every part of the health system. In these ways, the English experience since the 1990s sheds light on the development of health research systems worldwide.

## Data Availability

The datasets generated and analysed during the current study are not publicly available because this was a condition of the interviewees’ consent to participate. All of them were figures in public life and many wish to preserve the confidentiality of their statements about policy debates and controversies.

## Abbreviations

BIGT: Biosciences Innovation and Growth Team
CIHR: Canadian Institutes of Health Research
DoH: Department of Health
DHSC: Department of Health and Social Care
EBM: Evidence-based medicine
HTA: Health technology assessment
MRC: Medical Research Council
NHS: National Health Service
NICE: National Institute for Health and Care Excellence
NIHR: National Institute for Health Research
PICTF: Pharmaceutical Industry Competitiveness Task Force
R&D: Research and development
RDD: Research and Development Directorate (of the Department of Health)
ZonMw: (Netherlands) Organisation for Health Research and Development)
